# Split Intein-Mediated Engineering of Bacteriocins Through Backbone Circularization, Modular Assembly of Chimeras, and Inducible Circularization

**DOI:** 10.3390/biom16091311

**Published:** 2026-09-10

**Authors:** Irene Lafuente, Ester Sevillano, Nuria Peña, Cleopatra Collado, Luis M. Cintas, Pablo E. Hernández, Estefanía Muñoz-Atienza, Juan Borrero

**Affiliations:** Departamento de Nutrición y Ciencia de los Alimentos (NUTRYCIAL), Sección Departamental de Nutrición y Ciencia de los Alimentos (SD-NUTRYCIAL), Facultad de Veterinaria, Universidad Complutense de Madrid (UCM), Avenida Puerta de Hierro, s/n, 28040 Madrid, Spain; irelafue@ucm.es (I.L.); estsev01@ucm.es (E.S.); nuriapen@ucm.es (N.P.); cleopatra.collado@ucm.es (C.C.); lcintas@vet.ucm.es (L.M.C.); ehernan@vet.ucm.es (P.E.H.); jborrero@ucm.es (J.B.)

**Keywords:** in vitro cell-free protein synthesis (IV-CFPS), split intein-mediated ligation (SIML), protein trans-splicing, circular bacteriocins, chimeric bacteriocins, antimicrobial peptide engineering

## Abstract

The increasing prevalence of antimicrobial resistance has renewed interest in bacteriocins and other antimicrobial peptides as promising alternatives to conventional antibiotics. In this context, in vitro cell-free protein synthesis (IV-CFPS) provides a rapid and versatile platform for the production and functional evaluation of bacteriocins, enabling antimicrobial screening without the limitations associated with cellular expression, low production yields, or peptide toxicity. In previous studies, we demonstrated the utility of IV-CFPS for the production and characterization of a broad range of bacteriocins. More recently, integration of IV-CFPS with split intein-mediated ligation (SIML) enabled the production and functional evaluation of circular bacteriocins, thereby expanding the repertoire of bacteriocins accessible to cell-free synthesis and screening. Here, we further expand the capabilities of the IV-CFPS/SIML platform through three complementary bacteriocin engineering strategies. First, SIML was applied to the backbone circularization of naturally linear bacteriocins, demonstrating that SIML-mediated circularization can be extended beyond naturally circular bacteriocins. Second, the platform enabled the modular assembly of chimeric bacteriocins through the ligation of the translocation/receptor-binding (T-R) and cytotoxic (C) domains of either the same or different colicins. Finally, we developed a proof-of-concept inducible protein trans-splicing system in which bacteriocin circularization is triggered by the addition of a complementary split intein component, thereby providing temporal control over peptide activation. These results establish IV-CFPS/SIML as a versatile platform for programmable bacteriocin engineering and substantially expand its applications beyond the production of naturally circular bacteriocins.

## 1. Introduction

The rapid global emergence of antimicrobial resistance (AMR) has intensified the search for alternative antimicrobial strategies capable of complementing or replacing conventional antibiotics. Among these alternatives, bacteriocins have attracted considerable attention because of their potent antimicrobial activity, diverse mechanisms of action, and broad potential applications in food preservation, veterinary medicine, and human health [1,2,3]. Bacteriocins comprise a structurally diverse group of ribosomally synthesized antimicrobial peptides and proteins, ranging from small unmodified peptides and ribosomally synthesized and post-translationally modified peptides (RiPPs), predominantly produced by Gram-positive bacteria, to larger multidomain protein bacteriocins, such as colicins, produced by Gram-negative bacteria [2,4,5]. Although the discovery of novel bacteriocins through classical isolation procedures and genome mining remains an active area of research, increasing attention is now focused on engineering known bacteriocins to enhance their antimicrobial activity, stability, spectrum of activity, and other functional properties [6].

Numerous studies have demonstrated that rational engineering can generate bacteriocins with improved antimicrobial potency, altered target specificity, or enhanced physicochemical stability [7,8]. However, most engineering strategies continue to rely on conventional recombinant expression systems, which are often labor-intensive and technically demanding [6]. Many bacteriocins exhibit toxicity toward the expression host, require dedicated secretion or immunity systems, or are poorly compatible with heterologous production platforms, frequently resulting in low production yields or inactive products. In vitro cell-free protein synthesis (IV-CFPS) has emerged as a powerful alternative that overcomes many of these limitations by enabling the rapid production and functional evaluation of antimicrobial peptides independently of cellular constraints [9,10,11]. Previous studies have demonstrated the utility of IV-CFPS for the production and screening of large bacteriocin collections [6,10], as well as for the functional validation of bacteriocin precursor peptides identified through genome mining approaches [12,13,14,15,16]. More recently, the development of multiplexed IV-CFPS workflows has enabled the parallel production and high-throughput screening of large numbers of antimicrobial peptide candidates, further accelerating bacteriocin discovery and engineering [17].

Despite the versatility of IV-CFPS platforms, the production of many ribosomally synthesized and post-translationally modified peptides (RiPPs) remains challenging, particularly for molecules that require complex post-translational maturation. Circular bacteriocins represent one of such family of RiPPs. These peptides are characterized by a covalent head-to-tail linkage between their N- and C-termini, a structural feature that confers enhanced resistance to termal denaturation, pH variation, and proteolytic degradation, but also represents a major obstacle for in vitro production because efficient backbone circularization requires precise peptide ligation [18]. To overcome this limitation, we recently developed an IV-CFPS platform integrated with split intein-mediated ligation (SIML), enabling the production and functional evaluation of naturally circular bacteriocins through in vitro protein trans-splicing [12]. Inteins are self-splicing proteins that catalyze protein splicing, whereas split-inteins mediate protein trans-splicing through the highly specific ligation of independently produced protein fragments. Owing to their exceptional catalytic efficiency, specificity, and modularity, split inteins have emerged as powerful tools for synthetic biology and protein engineering [19].

Despite these advances, the broader potential of SIML as a platform for bacteriocin engineering has remained largely unexplored. In the present study, we expanded the capabilities of the IV-CFPS/SIML platform through three complementary engineering strategies designed to increase the diversity of antimicrobial architectures accessible to cell-free production and functional screening. Rather than focusing on the optimization of a single bacteriocin scaffold, we selected structurally and functionally diverse bacteriocins to evaluate the general applicability of the platform across different molecular sizes, topologies, and domain organizations. First, we investigated the feasibility of backbone circularization of naturally linear bacteriocins. Second, we evaluated the modular assembly of chimeric bacteriocins through protein trans-splicing of independently produced functional domains. Finally, we developed a proof-of-concept inducible protein trans-splicing system in which bacteriocin circularization is activated through hierarchical split intein reconstitution. Together, these approaches substantially broaden the scope of split intein-mediated bacteriocin engineering and establish IV-CFPS/SIML as a versatile platform for the rapid prototyping, production, and functional evaluation of engineered antimicrobial peptides, while providing a flexible framework for the future development of controlled activation and scalable production strategies.

## 2. Materials and Methods

### 2.1. Design and Construction of SIML Expression Platforms for Bacteriocin Engineering

To evaluate the applicability of split intein-mediated ligation (SIML) for bacteriocin engineering using an in vitro cell-free protein synthesis (IV-CFPS/SIML) platform, a series of expression constructs was designed for three independent applications: (i) backbone circularization of naturally linear bacteriocins, (ii) modular assembly of bacteriocin-derived functional domains, and (iii) development of an inducible SIML platform for the production and functional evaluation of circular bacteriocins. To assess the broad applicability of these engineering strategies, mature peptide sequences were selected from structurally and functionally diverse Gram-positive and Gram-negative bacteriocins encompassing different bacteriocin families. These included aureocin A53, enterocin A, enterocin HF, hiracin JM79, colicin M, microcin V, colicin Ib, colicin E6, colicin S4, colicin D, and salmocin E1B (Supplementary Data S1). In all constructs, nucleotide sequences encoding the mature bacteriocins were positioned between the N- and C-terminal fragments (NpuN and NpuC, respectively) of the *Nostoc punctiforme* DnaE (Npu DnaE) split intein to enable post-translational backbone circularization through protein trans-splicing [12]. Circularization sites were selected according to the presence of amino acid residues compatible with intein-mediated trans-splicing, primarily serine or cysteine residues, which serve as the nucleophile residue initiating the splicing reaction. When multiple potential ligation sites were present within a bacteriocin sequence, the final position was selected empirically based on sequence context and construct design considerations. The selected ligation residues were S509 for colicin Ib, S75 for colicin E6, S294 for colicin S4, S210 for colicin D, C51 for colicin M, C510 for salmocin E1B, C76 for microcin V, S2 for aureocin A53, S34 for enterocin A, S8 for enterocin HF, and C15 for hiracin JM79 (Supplementary Data S2).

Additional SIML constructs were designed to evaluate the modular assembly of bacteriocin-derived protein domains. For this purpose, the transport/receptor-binding (T-R) domains from colicin K and salmocin E1B, respectively, were independently fused to the N-terminal intein fragment (NpuN) of the Npu DnaE split intein, whereas their corresponding cytotoxic C-terminal (C) domains were fused to the C-terminal split intein fragment NpuC), enabling the generation of parental and chimeric bacteriocins following protein trans-splicing (Supplementary Data S3). An inducible protein trans-splicing system was also developed as a proof of concept to achieve conditional bacteriocin circularization through hierarchical split intein assembly. In this design, a truncated N-terminal fragment (C1-E27; NpuNA) of the Npu DnaE split intein was fused to the C-terminal fragment (CatC) of the Cat split intein [20], whereas the complementary construct consisted of the N-terminal Cat intein fragment (CatN) fused to the remaining portion (C28-T104; NpuNB) of the Npu DnaE N-terminal fragment (Supplementary Data S4). Following Cat intein-mediated protein trans-splicing, the complete NpuN fragment was reconstituted, thereby enabling a second trans-splicing reaction with NpuC to produce the circular bacteriocin. All SIML constructs were designed from published amino acid sequences and cloned into pUC-derived expression plasmids for T7 RNA polymerase-driven cell-free protein synthesis. Each expression cassette comprised a T7 promoter, an ATG start codon, a TAA stop codon, and a T7 transcription terminator. Gene synthesis and plasmid construction were performed by GeneArt™ (Thermo Fisher Scientific, Waltham, MA, USA).

### 2.2. IV-CFPS/SIML Protein Trans-Splicing, Antimicrobial Activity Assays, and Chemical Synthesis of Bacteriocins

Purified plasmid encoding the SIML constructs were used as templates for the in vitro cell-free protein synthesis at a final concentration of 10 ng/µL in 25 µL reaction volumes using the PURExpress^®^ In vitro Protein Synthesis Kit (New England Biolabs, Ipswich, MA, USA), according to the manufacturer’s instructions. Following protein synthesis, the reaction mixtures were incubated at room temperature for 24 h to promote split intein-mediated protein trans-splicing and, when applicable, backbone circularization. For modular ligation experiments involving colicin M and salmocin E1B domains, equal volumes of IV-CFPS/SIML reactions containing the transport/receptor-binding (T-R) domains and the cytotoxic (C) domains were mixed after protein synthesis and incubated at room temperature for 24 h to promote split-intein mediated trans-splicing and domain ligation. Similarly, equal volumes of IV-CFPS/SIML reactions containing the NpuC-EntAS48-NpuNA-CatN construct and the complementary CatC-NpuNB precursor were combined after protein synthesis and incubated at room temperature for 24 h to allow hierarchical split intein reconstitution, sequential protein trans-splicing, and bacteriocin backbone circularization.

The antimicrobial activity assays of the crude IV-CFPS/SIML reaction mixtures was evaluated using the spot-on-agar test (SOAT), as previously described [14]. The activity of constructs derived from Gram-negative bacteriocins, including the parental and chimeric colicin K/salmocin E1B proteins, was evaluated against *Escherichia coli* DH5α as the indicator strain. Briefly, 5 μL of each reaction mixture was spotted onto Luria–Bertani (LB) agar plates (1.5% *w*/*v*; Scharlab, Barcelona, Spain) overlaid with soft LB agar (0.8% *w*/*v*) inoculated with approximately 10^5^ CFU mL^−1^ of the indicator strain. The plates were incubated at 30 °C for 24 h, after which the presence of inhibition zones was recorded. The antimicrobial activity of constructs derived from Gram-positive bacteriocins, including those generated using the inducible platform circularization, was evaluated against *Pediococcus damnosus* CECT 4797. In these assays, de Man, Rogosa and Sharpe (MRS) agar plates (1.5% *w*/*v*; Condalab, Madrid, Spain) were overlaid with soft MRS agar (0.8% *w*/*v*) inoculated with approximately 10^5^ CFU mL^−1^ of the indicator strain. The plates were incubated at 30 °C for 24 h, and inhibition zones were subsequently recorded. When indicated the antimicrobial activity of selected IV-CFPS/SIML reaction mixtures was quantified using a microtiter plate assay (MPA). Growth inhibition of the indicator cultures was monitored spectrophotometrically at 620 nm using a FLUOstar OPTIMA microplate reader (BMGLabtech, Ortenberg, Germany). One bacteriocin unit (BU) was defined as the reciprocal of the highest dilution of the bacteriocin-containing sample that produced 50% growth inhibition relative to the untreated growth culture.

To enable a direct comparison of the antimicrobial activity of naturally linear and backbone- circularized bacteriocins variants independently of differences in IV-CFPS and IV-CFPS/SIML production yields, selected peptides were chemically synthesized by GenScript Biotech Ltd. (Oxford, UK). Linear enterocin A and aureocin A53 were successfully synthesized at purities exceeding 95%. In addition, a head-to-tail backbone-circularized form of enterocin A was successfully synthesized, whereas attempts to synthesize a circular form of aureocin A53 were unsuccessful. The antimicrobial activities of the chemically synthesized linear and circular enterocin A variants were evaluated using the SOAT. Starting from an initial peptide concentration of 0.5 mg mL^−1^, 0.5 µL aliquots of two-fold serial dilutions were spotted onto MRS agar plates overlaid with soft MRS agar (0.8% *w*/*v*) inoculated with approximately 10^5^ CFU mL^−1^ of *Listeria monocytogenes* CECT 4032 as the indicator strain. The plates were incubated at 30 °C for 24 h, after which inhibition zones were recorded.

## 3. Results and Discussion

Bacteriocins have attracted increasing interest as promising alternatives to conventional antibiotics owing to their potent antimicrobial activity, diverse mechanisms of action, and generally narrow target spectra. Because they are ribosomally synthesized peptides or proteins, many bacteriocins are amenable to synthetic biology approaches, particularly class II bacteriocins, which typically require little or no post-translational modifications. In recent years, in vitro cell-free protein synthesis (IV-CFPS) has emerged as a powerful platform for the rapid production and functional evaluation of proteins and peptides independently of cellular expression constraints.

Compared with conventional heterologous expression systems, IV-CFPS offers several advantages, including the elimination of host toxicity, simplified reaction control, rapid prototyping, and straightforward scalability. Although its application to bacteriocin discovery and production remains relatively limited [10], we previously demonstrated that IV-CFPS enables the rapid production and functional validation of bacteriocins identified by genome mining [12,13,14,15,16]. More recently, we expanded the platform to support the multiplexed synthesis of bacteriocin combinations and antimicrobial cocktails targeting multidrug-resistant bacteria [17]. Beyond peptide production, IV-CFPS also provides a versatile environment for post-translational peptide engineering. By exploiting the ability of split inteins to catalyze highly specific protein trans-splicing reactions, the IV-CFPS/SIML platform established a simple and versatile strategy for the in vitro generation of circular antimicrobial peptides without requiring their native biosynthetic machineries [12,14].

Split inteins are versatile tools for antimicrobial peptide engineering because they enable the covalent assembly of independently produced peptide and protein fragments through highly specific protein trans-splicing reactions. In addition to peptide backbone circularization, they can facilitate the modular recombination of functional domains, thereby extending their utility beyond the production of naturally circular bacteriocins. Accordingly, the present study further explores three complementary applications of the IV-CFPS/SIML platform for bacteriocin engineering: (i) backbone circularization of naturally linear bacteriocins, (ii) modular assembly of chimeric colicins through post-expression ligation of independently produced functional domains, and (iii) development of an inducible protein trans-splicing system based on hierarchical split intein reconstitution for controlled bacteriocin circularization. These approaches were designed to evaluate the versatility of SIML-mediated assembly as a programmable strategy for expanding the molecular engineering of bacteriocins.

### 3.1. SIML-Mediated Backbone Circularization of Naturally Linear Bacteriocins

Circular bacteriocins constitute one of the most structurally robust groups of ribosomally synthesized and post-translationally modified antimicrobial peptides (RiPPs), typically exhibiting greater resistance to thermal denaturation, pH fluctuations, and proteolytic degradation than their linear counterparts. These enhanced physicochemical properties are primarily attributed to their head-to-tail backbone circularization, which reduces conformational flexibility while protecting the peptide from exopeptidase-mediated degradation. Based on these characteristics, we hypothesized that extending SIML-mediated backbone circularization to naturally linear bacteriocins could provide a general strategy for generating engineered antimicrobial peptides with improved physicochemical stability and robustness. To test this hypothesis across a broad range of bacteriocin architectures, we deliberately selected structurally diverse bacteriocins representing different families and molecular sizes.

**Figure 1:**
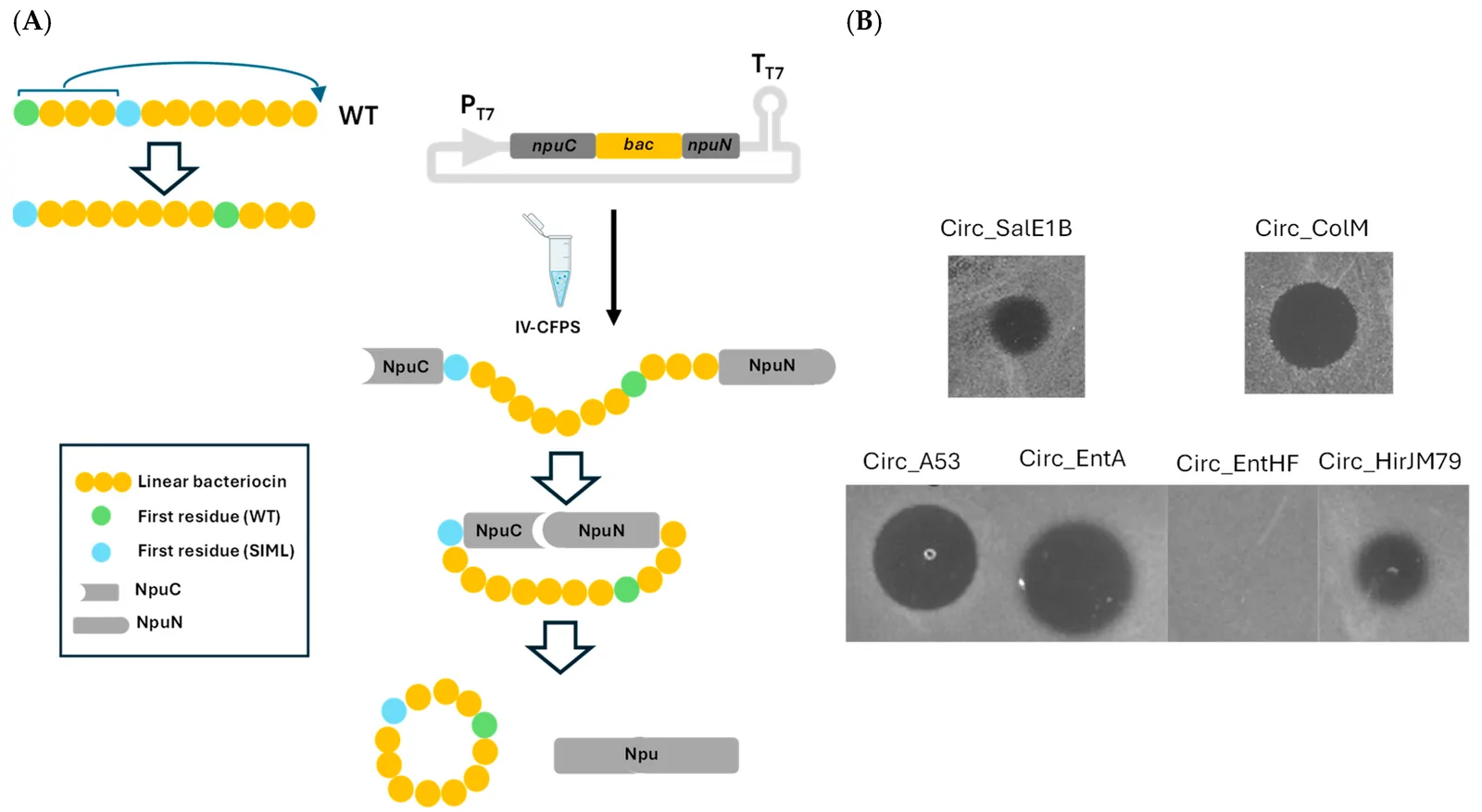
SIML-mediated backbone circularization of naturally linear bacteriocins and antimicrobial activity of the resulting circular variants. (**A**) Schematic representation of the strategy used to generate backone-circularized variants of naturally linear bacteriocins using split intein-mediated ligation (SIML). An internal serine or cysteine residue within the mature peptide sequence was selected as the +1 nucleophilic residue required to initiate protein trans-splicing. The mature peptide sequence was then rearranged so that the selected residue occupied the ligation junction while preserving its native amino acid composition. The rearranged peptide sequence was flanked by the N- and C-terminal fragments (NpuC and NpuN) of the *Nostoc punctiforme* DnaE split intein, enabling protein trans-splicing, peptide ligation, and head-to-tail backbone circularization following IV-CFPS/SIML. (**B**) Antimicrobial activity of the backbone-circularized bacteriocins produced by IV-CFPS/SIML. The upper panels show the activity of the circular salmocin E1B (Cir_SalE1B) and colicin M (Cir_ColM) variants against *Escherichia coli* DH5α. The lower panels show the activity of the circular aureocin A53 (Cir_ A53), enterocin A (Cir_EntA), enterocin HF (Cir_EntHF), and hiracin JM79 (Cir_HirJM79) variants against *Pediococcus damnosus* CECT 4797.

Accordingly, we investigated whether SIML-mediated backbone circularization is compatible with the antimicrobial activity of bacteriocins that are naturally produced as linear peptides. The construct design was based on our previously described IV-CFPS/SIML platform for the production of circular bacteriocins (Figure 1A) [12,14], in which the native mature peptide sequences were replaced with naturally linear antimicrobial peptides. Specifically, the mature peptide sequences of colicins S4, E6, Ib, D, and M, the colicin-like bacteriocin salmocin E1B, microcin V, the class II bacteriocins enterocin A, hiracin JM79, and enterocin HF, and the leaderless aureocin A53 were individually cloned between the N- and C-terminal fragments (NpuC and NpuN) of *Nosctoc puntiforme* DnaE split intein and expressed using the IV-CFPS/SIML platform. For each bacteriocin, an internal serine or cysteine residue was selected as the +1 nucleophilic residue required to initiate SIML-mediated protein trans-splicing. The mature peptide sequence was rearranged so that the selected residue occupied the ligation junction, while preserving the original amino acid composition. This design enabled head-to-tail backbone circularization of each bacteriocin without introducing amino acid substitutions into the mature peptide sequence.

Following IV-CFPS production, antimicrobial activity assays revealed that only a subset of the engineered bacteriocins retained detectable activity after SIML-mediated backbone circularization (Figure 1B). Among the Gram-negative bacteriocins, only the circularized variants of salmocin E1B and colicin M remained active, whereas all other colicin-derived constructs were inactive. Similarly, among the naturally linear class II bacteriocins, the circularized variants of aureocin A53, enterocin A, and hiracin JM79 retained antimicrobial activity, whereas no activity was detected for the circularized enterocin HF.

The different outcomes observed across this structurally diverse bacteriocin collection indicate that the compatibility of SIML-mediated backbone circularization with antimicrobial activity is bacteriocin-dependent, with activity being retained only by a subset of naturally linear bacteriocins. However, the absence of or reduction in antimicrobial activity should not be interpreted as unequivocal evidence of unsuccessful circularization or of an inherent incompatibility between a given bacteriocin and a circular topology. Because antimicrobial activity was evaluated directly from the IV-CFPS/SIML reaction mixtures, the absence of detectable activity could result from inefficient protein production, incomplete protein trans-splicing/circularization, or loss of antimicrobial activity of the resulting circular product. In this regard, the efficiency of SIML-mediated protein trans-splicing is strongly influenced by the amino acid residues flanking the ligation junction, particularly those at the −1/+1 positions, as well as by the local structural environment of the mature peptide, all of which can markedly affect protein trans-splicing efficiency and the yield of correctly circularized products [21,22,23]. Consequently, lack of activity alone cannot be considered evidence that a particular bacteriocin is incompatible with backbone circularization. Further analytical characterization of individual IV-CFPS/SIML products will therefore be required to determine protein trans-splicing efficiency and quantify the correctly circularized products.

These considerations are likely to be particularly relevant for the larger bacteriocins salmocin E1B and colicin M. Whereas IV-CFPS of the corresponding linear constructs is expected to produce predominantly full-length, biologically active proteins, IV-CFPS/SIML reactions are more likely to generate heterogeneous product mixtures containing un-spliced precursors, partially processed intermediates, and correctly circularized bacteriocins. Accordingly, the antimicrobial activity detected in the circularized constructs is expected to reflect both the efficiency of the SIML-mediated reaction and the intrinsic activity of the circularized bacteriocin, rather than the biological properties of the circular molecule alone.

**Figure 2:**
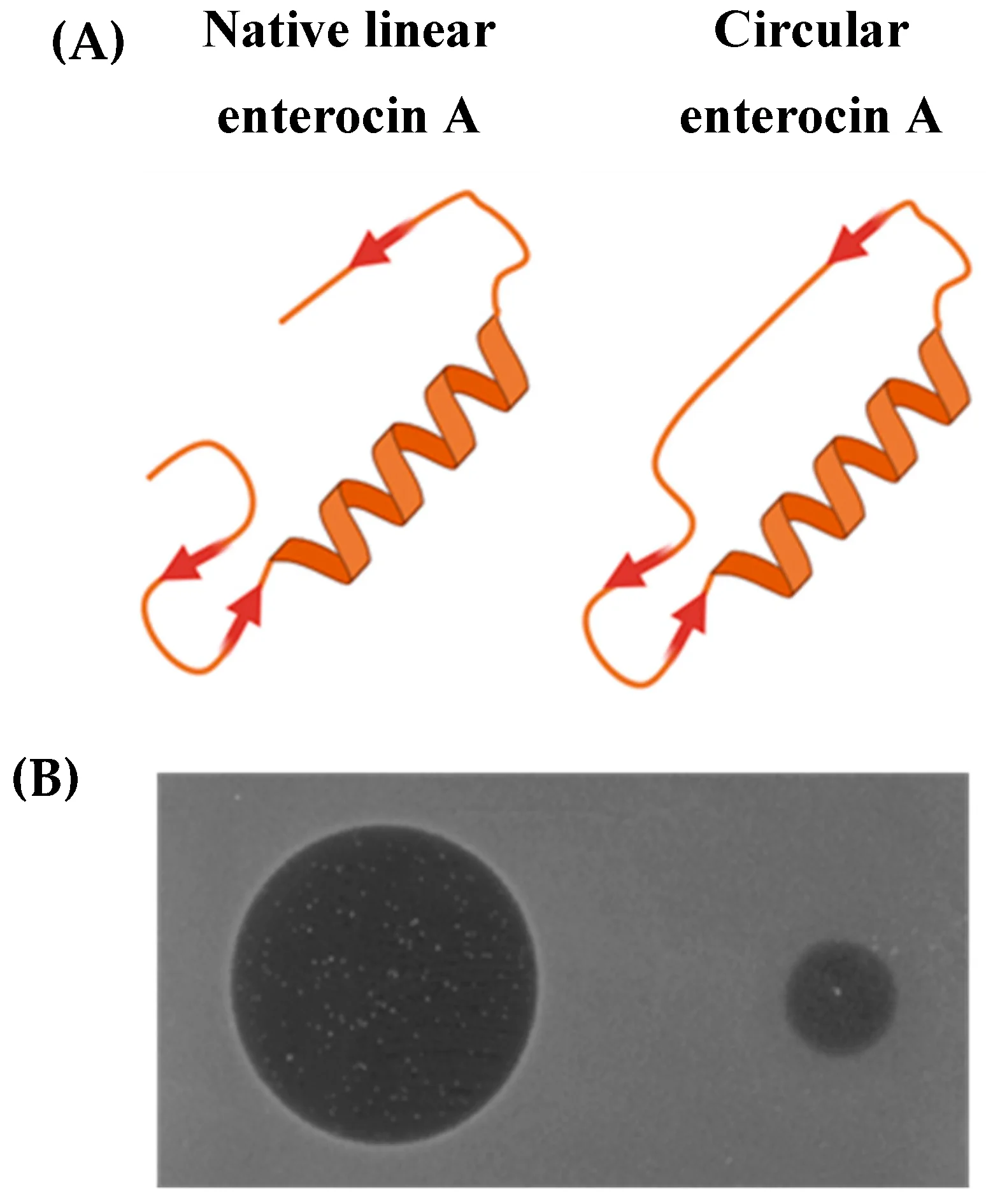
Topological representation and antimicrobial activity of chemically synthesized linear and backbone-circularized enterocin A. (**A**) Schematic representations of the linear and backbone-circularized forms of enterocin A. The diagrams are intended solely to illustrate the peptide topology and do not represent experimentally determined or computationally predicted three-dimensional structures. (**B**) Antimicrobial activity of the chemically synthesized linear (**left**) and the backbone-circularized enterocin A (**right**) enterocin A variants, determined by the spot-on-agar test (SOAT) using *L. monocytogenes* CECT 4032 as the indicator strain at a peptide concentration of 0.5 mg mL^−1^.

To further address these observations, we compared the antimicrobial activities of chemically synthesized naturally linear bacteriocins and their corresponding backbone-circularized variants using homogeneous peptide preparations at equivalent concentrations. This approach enabled the intrinsic antimicrobial activities of the linear and circular peptides to be evaluated independently of the efficiency of the IV-CFPS/SIML reaction. Because of their relatively long amino acid sequences and large molecular masses, the chemical synthesis of the naturally linear and circular variants of salmocin E1B and colicin M was not feasible. Likewise, attempts to chemically synthesize the circular forms of aureocin A53 and hiracin JM79 were unsuccessful. In contrast, both the naturally linear and backbone-circularized forms of enterocin A were successfully synthesized and purified. Comparative antimicrobial activity assays revealed a marked reduction in the activity of the circular enterocin A relative to its naturally linear counterpart, as evidenced by the loss of inhibitory activity after substantially fewer two-serial dilutions (Figure 2 and Figure S1). These findings suggest that head-to-tail backbone circularization may compromise structural and/or functional features required for the optimal antimicrobial activity of enterocin A.

The reduced antimicrobial activity observed for circular enterocin A most likely reflects the conformational constraints imposed by head-to-tail backbone circularization. Unlike naturally circular bacteriocins, whose structures have evolved to accommodate a cyclic backbone, many naturally linear bacteriocins may require a certain degree of terminal flexibility to achieve optimal membrane interaction, receptor recognition, or pore formation. Consequently, backbone circularization may alter peptide conformational dynamics, folding, or the spatial presentation of residues that are critical for antimicrobial activity.

Despite the reduction in intrinsic antimicrobial potency, the enhanced physicochemical stability generally associated with circular peptides may still provide functional advantages. Although these properties were not experimentally evaluated in the present study, future studies should evaluate whether the reduced intrinsic activity of engineered circular variants could be compensated by improved peptide stability, prolonged activity, or enhanced performance under harsh environmental or industrial conditions, including exposure to elevated temperatures, extreme pH values, or proteolytic environments. Additionally, these findings demonstrate that the IV-CFPS/SIML platform can be extended beyond the production of naturally circular bacteriocins and highlight the potential of SIML-mediated backbone circularization as a versatile strategy for engineering bacteriocin derivatives with tailored structural and functional properties.

### 3.2. SIML-Mediated Assembly of Chimeric Bacteriocins Through Modular Ligation of Functional Domains

In a previous study, we demonstrated that chimeric colicins could be generated by genetically recombining the translocation/receptor-binding (T-R) and catalytic (C) domains from different colicins, yielding engineered bacteriocins with altered or expanded antimicrobial spectra [14]. These findings highlighted the highly modular organization of colicins and established domain recombination as a promising strategy for the development of next-generation protein bacteriocins targeting Gram-negative bacteria. However, the generation of these chimeric constructs relied on conventional recombinant DNA assembly and PCR-based cloning, approaches that become increasingly laborious as the number of domain combinations increases. We therefore investigated whether SIML-mediated protein trans-splicing could provide a more flexible platform for assembling chimeric bacteriocins through the post-translational ligation of independently synthesized functional domains.

**Figure 3:**
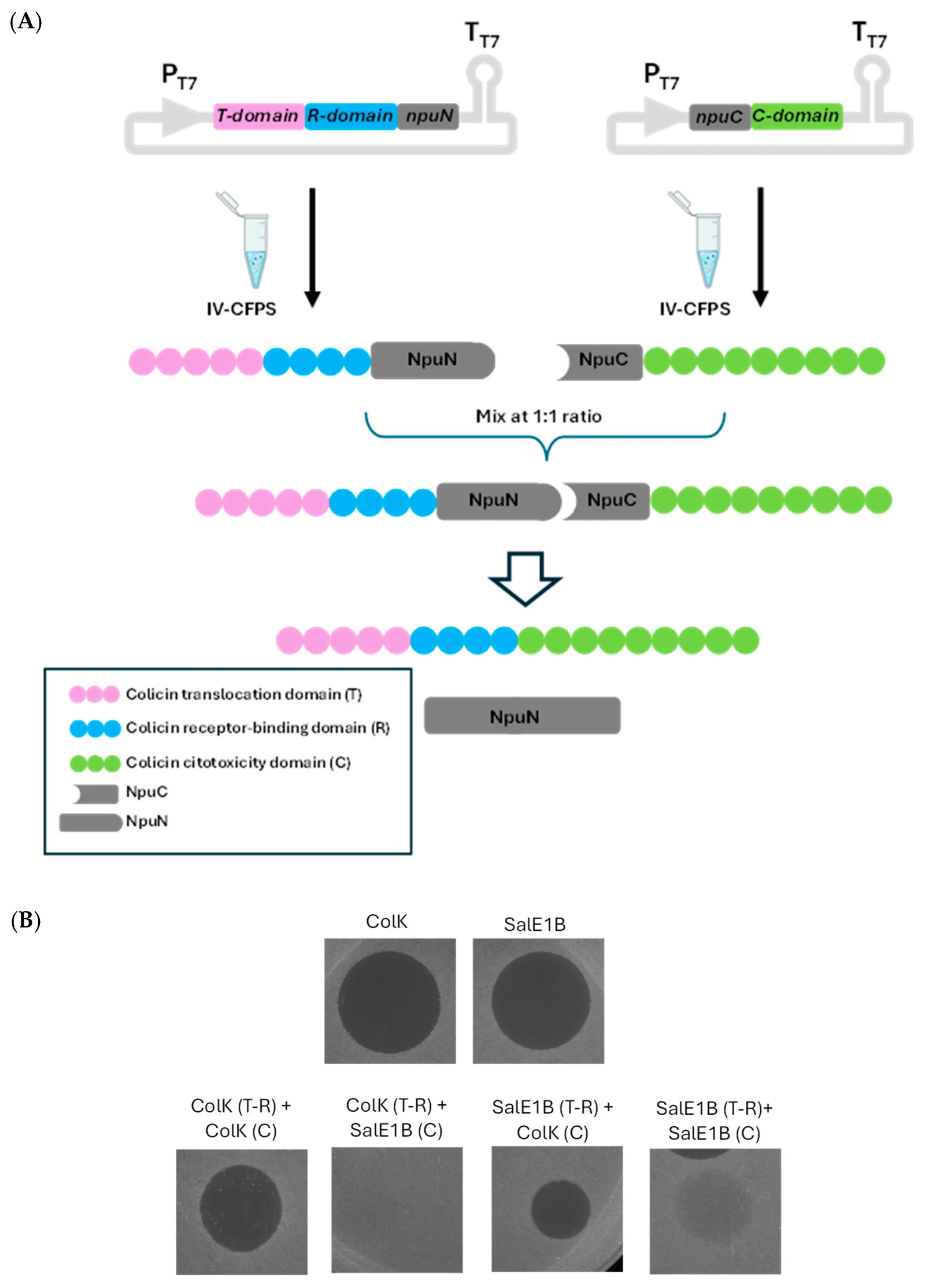
IV-CFPS/SIML-mediated assembly of colicin-derived chimeric bacteriocins through modular ligation of translocation/receptor binding (T-R) and cytotoxic (C) domains. (**A**) Schematic representation of the IV-CFPS/SIML strategy used to generate colicin-derived chimeric bacteriocins by split intein-mediated protein trans-splicing and modular ligation of independently synthesized T-R and C domains. (**B**) Antimicrobial activity of IV-CFPS-produced full-length naturally linear bacteriocins (**upper panel**) and the parental and chimeric bacteriocins assembled by IV-CFPS/SIML (**lower panel**), evaluated against *Escherichia coli* DH5α.

To evaluate this strategy, the translocation/receptor-binding (T-R) and cytotoxic (C) domains of colicin K (ColK) and salmocin E1B (SalE1B) were selected as model scaffolds because both bacteriocins share the canonical modular organization of pore-forming colicins, making them well-suited for evaluating split intein-mediated domain recombination. The T-R domains were fused to the N-terminal fragment (NpuN), whereas the C domains were fused to the complementary C-terminal fragment (NpuC) of the *Nostoc punctiforme* DnaE split intein. Following independent synthesis by IV-CFPS, the corresponding reaction mixtures were combined to promote split intein-mediated protein trans-splicing and covalent ligation of the T-R and C domains, thereby generating full-length parental and chimeric bacteriocins (Figure 3A). This modular strategy enabled domain swapping between the T-R and C domains of ColK and SalE1B without the need to construct a new expression plasmid for each chimeric combination. For comparison, the corresponding full-length linear ColK and SalE1B bacteriocins were also produced by IV-CFPS and used as reference molecules for evaluating the antimicrobial activity of the IV-CFPS/SIML-assembled constructs.

Following independent IV-CFPS production of the split intein-fused (SIML) precursor fragments and subsequent protein trans-splicing, antimicrobial assays confirmed that the reconstituted parental combinations, ColK (T-R) + ColK (C) and SalE1B (T-R) + SalE1B (C), retained detectable antimicrobial activity, although the inhibition halos were smaller than those produced by the corresponding full-length naturally linear bacteriocins, particularly in the case of SalE1B (Figure 3B). Among the chimeric constructs only the SalE1B (T-R) + ColK (C) combination exhibited detectable antimicrobial activity, whereas the reciprocal chimera, ColK (T-R) + SalE1B (C), failed to inhibit the growth of the indicator strain under the conditions tested.

The reduced antimicrobial activity observed for the SIML-assembled bacteriocins most likely reflects intrinsic limitations associated with protein trans-splicing-mediated assembly. Unlike naturally linear bacteriocins, which are synthesized directly as full-length proteins, the engineered constructs depend on the efficient ligation of independently synthesized precursor fragments. Thus, incomplete protein trans-splicing is expected to generate heterogeneous reaction mixtures containing un-ligated precursors, partially processed intermediates, and correctly assembled bacteriocins, thereby reducing the proportion of biologically active molecules. Moreover, protein trans-splicing efficiency is strongly influenced by the amino acid residues flanking the ligation junction, particularly the −1 and +1 extein residues, which are known to modulate split intein catalytic activity [24]. The folding and conformational state of the independently synthesized T-R and C domains before ligation may further influence split intein accessibility, trans-splicing efficiency, and the subsequent folding of the reconstituted bacteriocin, as previously reported for other split intein-mediated protein assembly systems [24]. These factors are likely to be particularly important for the assembly of large multidomain bacteriocins such as colicins, whose antimicrobial activity depends on the coordinated structural organization and functional interplay of their translocation, receptor-binding, and cytotoxic domains.

Similarly, the different outcomes obtained for the reciprocal chimeric constructs indicate that the success of SIML-mediated domain recombination is strongly dependent on the structural and functional compatibility of the individual bacteriocin domains. The absence of detectable antimicrobial activity in the ColK (T-R) + SalE1B (C) chimera further indicates that not all modular domain combinations are compatible, despite the intrinsically modular organization of colicins. Although SIML-mediated protein trans-splicing enables the covalent ligation of translocation/receptor-binding (T-R) and cytotoxic (C) domains, successful ligation alone is insufficient to ensure biological activity. Functional compatibility between the assembled domains is likely required to achieve correct folding, receptor recognition, cellular translocation, and cytotoxic activity within susceptible bacteria.

Nevertheless, these findings demonstrate that split inteins can serve as efficient molecular tools for the modular assembly of multidomain bacteriocins. Compared with conventional recombinant DNA-based engineering strategies, SIML-mediated assembly greatly simplifies the generation and evaluation of combinatorial bacteriocin architectures by enabling the independent production of precursor fragments followed by their programmable in vitro ligation. This strategy provides a flexible and scalable platform for rapidly generating large libraries of engineered bacteriocins incorporating diverse translocation, receptor-binding, and cytotoxic domains. Such combinatorial diversity could facilitate the development of novel antimicrobial repertoires, including bacteriocin cocktails designed to minimize the emergence of resistance by simultaneously targeting multiple uptake pathways and distinct intracellular targets.

### 3.3. Development of an Inducible SIML Platform for Temporally Controlled Bacteriocin Circularization

Although the IV-CFPS/SIML platform provides a rapid and versatile approach for the synthesis and functional screening of circular bacteriocins, one of its principal limitations is the relatively low peptide yield that may be achieved with the reconstituted PURE system used in this study, together with the high reagent costs and limited reaction volumes [25,26]. These limitations should not, however, be generalized to all cell-free protein synthesis platforms, as protein yield and scalability can vary considerably depending on the CFPS system employed [25,26]. To overcome these limitations and facilitate larger-scale peptide production, we previously adapted the SIML platform for recombinant expression in *E. coli* [12,14]. This strategy enabled the in vivo production and purification of circular bacteriocins from recombinant cultures. However, induction of recombinant expression consistently resulted in severe growth inhibition, indicating that intracellular SIML-mediated circularization generated biologically active bacteriocins that were toxic to the producing host.

These observations identify a major bottleneck in the heterologous production of highly active circular bacteriocins using the SIML platform. Premature intracellular circularization is likely to generate active bacteriocins before sufficient biomass or inactive precursor proteins have accumulated, thereby limiting overall production yields. Introducing temporal control over SIML-mediated protein trans-splicing therefore represents an attractive strategy to uncouple precursor synthesis from bacteriocin circularization, minimizing intracellular toxicity while potentially improving production efficiency. Several approaches for regulating protein trans-splicing have previously been described, including systems controlled by temperature, small molecules, salt, light, proteases, and genetically encoded non-canonical amino acids [27,28,29,30]. Inspired by these strategies, we sought to develop an inducible SIML platform in which bacteriocin circularization is triggered only after the synthesis and accumulation of inactive precursor proteins, thereby providing temporal control over peptide activation.

**Figure 4:**
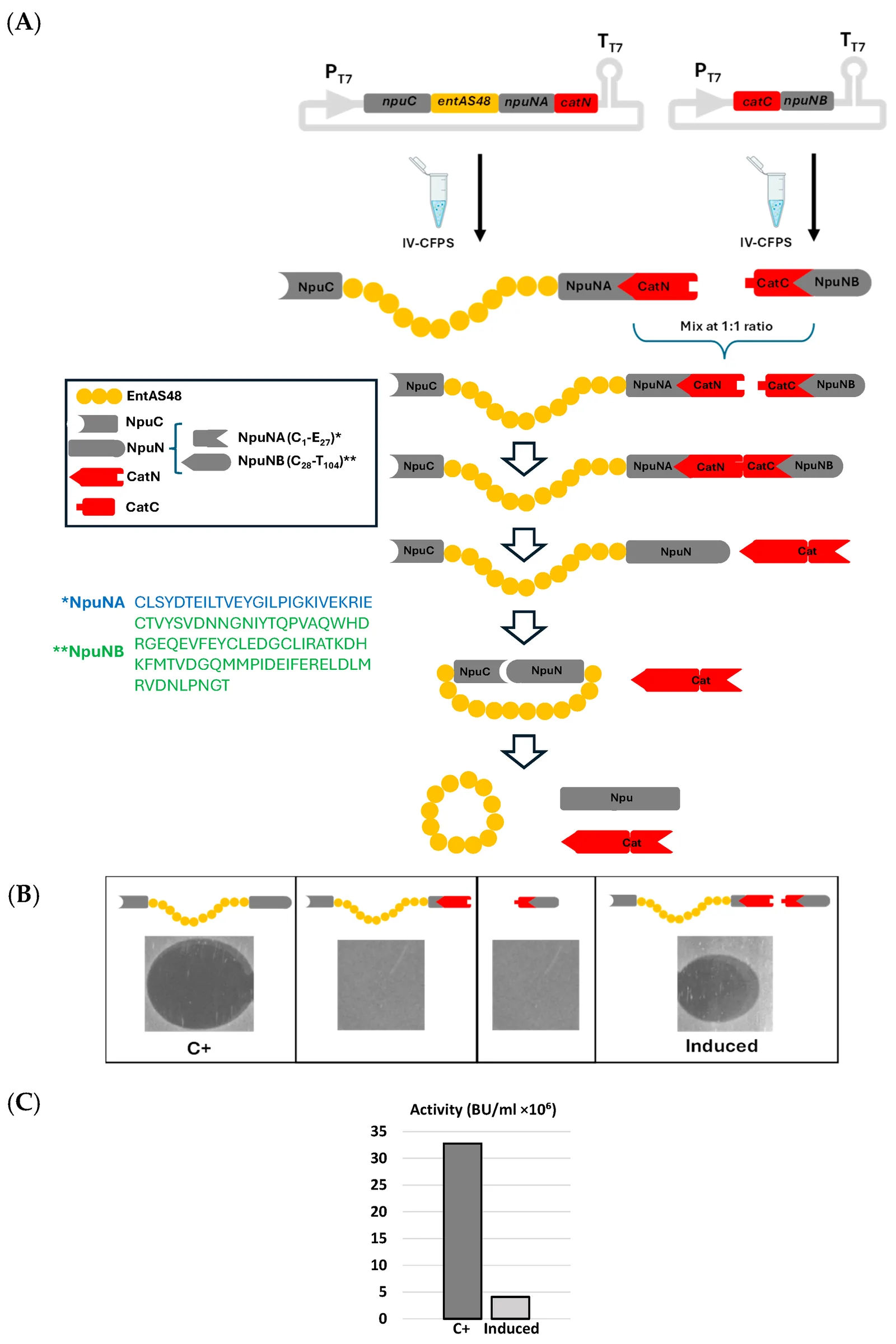
Development and functional validation of an inducible SIML platform for controlled bacteriocin circularization. (**A**) Schematic representation of the inducible SIML strategy based on the sequential reconstitution of two split inteins, enabling temporally controlled head-to-tail bacteriocin circularization through two consecutive protein trans-splicing reactions. (**B**) Antimicrobial activity of the individual precursor constructs and of the complete inducible SIML system following mixing of the IV-CFPS reaction products, evaluated against *Pediococcus damnosus* CECT 4797. (**C**) Comparison of the antimicrobial activity of the constitutive IV-CFPS/SIML platform (positive control, C^+^) and the inducible SIML system. Antimicrobial activity is expressed as bacteriocin units per milliliter (BU/mL).

To implement this strategy, we designed a two-step intein activation system based on the sequential reconstitution of the split inteins that drive SIML-mediated bacteriocin circularization. The underlaying rationale was to maintain the bacteriocin precursor in a catalytically inactive, non-splicing state during synthesis and to trigger circularization only after controlled assembly of the intein machinery (Figure 4A).

The design was based on the *Nostoc punctiforme* DnaE (Npu DnaE) split intein previously used for SIML-mediated bacteriocin circularization [12,14]. However, instead of incorporating the complete NpuN fragment, only its N-terminal 27 amino acid residues (C1-E27) were retained in the primary precursor construct. This truncated NpuN segment was fused to the N-terminal fragment of a second split intein, Cat (CatN) [20]. Because the NpuN fragment was incomplete, productive association with the complementary NpuC fragment could not occur, thereby preventing spontaneous protein trans-splicing and premature bacteriocin circularization.

A second precursor construct was engineered by fusing the C-terminal fragment of the Cat split intein (CatC) to the remaining portion of the NpuN intein (residues C28–T104). Upon mixing the two precursor constructs, CatN and CatC associate and catalyze the first protein trans-splicing reaction, resulting in covalent ligation and reconstitution of the complete NpuN fragment. The reconstituted NpuN fragment can then associate with the complementary NpuC fragment present in the SIML construct, initiating a second protein trans-splicing reaction that drives head-to-tail bacteriocin circularization. This design establishes a hierarchical intein cascade in which Npu DnaE-mediated bacteriocin circularization depends on the prior reconstitution of NpuN by the Cat split intein. By temporally separating intein reconstitution from bacteriocin circularization, this strategy provides an additional level of control over peptide activation, ensuring that the active antimicrobial peptide is generated only after completion of the initial assembly step.

To validate this strategy, the construct encoding Cat split intein-mediated reconstitution of NpuN and the corresponding enterocin As-48 circularization construct were produced independently using the IV-CFPS platform and evaluated for antimicrobial activity. Importantly, neither construct exhibited detectable antimicrobial activity when tested individually (Figure 4B), confirming that neither spontaneous Npu DnaE-mediated protein trans-splicing nor bacteriocin circularization occurred in the absence of Cat-mediated Npu reconstitution. In contrast, combining the two IV-CFPS reaction products resulted in clear antimicrobial activity, demonstrating successful Cat-mediated reconstitution of the complete NpuN fragment, followed by Npu DnaE-dependent protein trans-splicing and head-to-tail bacteriocin circularization (Figure 4B).

The inducible SIML platform exhibited approximately an eight-fold reduction in antimicrobial activity compared with the constitutive IV-CFPS-SIML system (Figure 4C). This decrease most likely reflects the requirement for two sequential protein trans-splicing reactions before formation of the active circular bacteriocin, thereby reducing the overall efficiency of the assembled process. In addition, incomplete intein reconstitution, steric constraints, or suboptimal folding of intermediate protein assemblies may further compromise trans-splicing efficiency.

## 4. Conclusions

In this work, we substantially expanded the scope of split intein-mediated ligation (SIML) as a versatile platform for antimicrobial peptide engineering by developing three complementary strategies within an in vitro cell-free protein synthesis (IV-CFPS) framework. First, SIML enabled the backbone circularization of naturally linear bacteriocins, demonstrating that SIML-mediated circularization can be extended beyond naturally circular bacteriocins to generate engineered variants with potentially enhanced physicochemical properties. Second, split inteins were successfully exploited for the modular assembly of chimeric bacteriocins through the ligation of independently produced functional domains, highlighting their potential as programmable molecular connectors for combinatorial bacteriocin engineering. Finally, we developed an inducible SIML system that enables temporally controlled bacteriocin circularization, providing a proof-of-concept for controlled split intein reconstitution as a strategy to facilitate heterologous production while minimizing intracellular toxicity.

Although further optimization is required to improve protein trans-splicing efficiency, ligation junction design, and preservation of antimicrobial activity following engineering, the heterogeneous outcomes observed across the different bacteriocin scaffolds highlight that the success of SIML-mediated bacteriocin engineering is inherently bacteriocin-dependent. Accordingly, future applications of the platform will benefit from integrating structural and mechanistic knowledge into construct design to guide the selection of engineering strategies best suited for individual bacteriocin scaffolds. The approaches described here considerably broaden the potential applications of split intein-mediated assembly in bacteriocin research. More broadly, the integration of IV-CFPS with SIML provides a rapid and flexible framework for the prototyping, modular assembly, and functional evaluation of engineered bacteriocins, thereby accelerating the development of next-generation cyclic antimicrobial peptides, multidomain protein bacteriocins, and other engineered peptide therapeutics.

## Data Availability

The data presented in this study are available within the article and its Supplementary Materials. Additional data are available from the corresponding author upon reasonable request.

## Supplementary Materials

The following supporting information can be downloaded at: https://www.mdpi.com/article/10.3390/biom16091311/s1, Supplementary Data S1: Bacteriocins analyzed in this study, their source organisms, and the corresponding UniProt accession numbers. Supplementary Data S2: Amino acid sequences of the SIML constructs used for bacteriocin backbone circularization. Supplementary Data S3: Amino acid sequences of the split intein constructs used for the modular assembly of colicin-derived bacteriocins. Supplementary Data S4: Amino acid sequences of the constructs used in the inducible SIML system. Supplementary Figure S1: Comparative antimicrobial activity of chemically synthesized backbone-circularized and native linear enterocin A across serial two-fold dilutions.
