# Supplementary material for: Split Intein-Mediated Engineering of Bacteriocins Through Backbone Circularization, Modular Assembly of Chimeras, and Inducible Circularization
Source: Biomolecules. 2026 Sep 10;16(9):1311. doi: 10.3390/biom16091311 (PMC13604234; doi:10.3390/biom16091311)
Supplement: Supplementary file 1 [file biomolecules-16-01311-s001.zip › biomolecules-4495152-supplementary.pdf]

Supplementary Data S1. Bacteriocins analyzed in this study, their source organisms, and the corresponding UniProt accession numbers.

**Bacteriocin**	**Source organism**	**Uniprot Entry**
Colicin M	*Escherichia coli*	A0A0H2XJY7
Microcin V	*Escherichia coli*	A0A0H3ER93
Colicin Ib	*Escherichia coli*	P04479
Colicin E6	*Escherichia coli*	P17999
Colicin S4	*Escherichia coli*	Q9XB47
Colicin D	*Escherichia coli*	P17998
Colicin K	*Escherichia coli*	Q47502
Salmocin E1B	*Salmonella enterica*	A0A0Q9Z932
Aureocin A53	*Staphylococcus aureus*	Q8GPI4
Enterocin A	*Enterococcus faecium*	I3U4Q4
Enterocin HF	*Enterococcus faecium*	P86183
Hiracin JM79	*Enterococcus hirae*	Q0Z8B6
Enterocin AS-48	*Enterococcus faecalis*	Q47765

Supplementary Data S2. Amino acid sequences of the SIML constructs used for bacteriocin backbone circularization. For clarity, the residues involved in the ligation junction are highlighted throughout the sequences. The nucleophilic residue (Ser or Cys) selected as the +1 residue for protein trans-splicing is shown in cyan, whereas the original N-terminal residue of the mature bacteriocin is highlighted in green. The N- and C-terminal fragments of the Npu DnaE split intein (NpuN and NpuC, respectively) are shown in grey.

Colicin Ib

Mature amino acid sequence (WT)

```
  1 MSDPVRITNP GAESLGYDSD GHEIMAVDIY VNPPRVDVFH GTPPAWSSFG NKTIWGGNEW
 61 VDDSPTRSDI EKRDKEITAY KNTLSVQQKE NENKRTEAGK RLSAAIAARE KDENTLKTLR
121 AGNADVADIT RQEFRLLQAE LREYGFRTEI AGYDALRLHT ESRMLFADAD SLRISPREAR
181 SLIEQAEKRQ KDAQNADKKA ADMLAEYERR KGILDTRLSE LEKNGGAALA VLDAQQARLL
241 GQQTRNDRAI SEARNKLSSV TESLKTARNA LTRAEQQLTQ QKNTPDGKTI VSPEKFPGRS
301 STNHSIVVSG DPRFAGTIKI TTSAVIDNRA NLNYLLTHSG LDYKRNILND RNPVVTEDVE
361 GDKKIYNAEV AEWDKLRQRL LDARNKITSA ESAVNSARNN VSARTNEQKH ANDALNALLK
421 EKENIRSQLA DINQKIAEEK RKRDEINMIK DAIKLTSDFY RTIYDEFGKQ ASELAKELAS
481 VSQGKQIKSV DDALNAFDKF RNNLNKKYSI QDRMAISKAL EAINQVHMAE NFKLFSKAFG
541 FTGKVIDRYD VAVELQKAVK TDNWRPFFVK LESLAAGRAA SAVTAWTFSV MLGTPVGILG
601 FAIIMAAVSA FVNDKFIEQV NKLIGI
```

SIML amino acid sequence

```
  1 MIKIATRKYL GKQNVYDIGV ERYHNFALKN GFIASNSIQD RMAISKALEA INQVHMAENF
 61 KLFSKAFGFT GKVIDRYDVA VELQKAVKTD NWRPFFVKLE SLAAGRAASA VTAWTFSVML
121 GTPVGILGFA IIMAAVSAFV NDKFIEQVNK LIGIMSDPVR ITNPGAESLG YDSDGHEIMA
181 VDIYVNPPRV DVFHGTPPAW SSFGNKTIWG GNEWVDDSPT RSDIEKRDKE ITAYKNTLSV
241 QQKENENKRT EAGKRLSAAI AAREKDENTL KTLRAGNADV ADITRQEFRL LQAELREYGF
301 RTEIAGYDAL RLHTESRMLF ADADSLRISP REARSLIEQA EKRQKDAQNA DKKADMLAE
361 YERRKGILDT RLSELEKNGG AALAVLDAQQ ARLLGQQTRN DRAISEARNK LSSVTESLKT
421 ARNALTRAEQ QLTQQKNTPD GKTIVSPEKF PGRSSTNHSI VVSGDPRFAG TIKITTSAVI
481 DNRANLNYLL THSGLDYKRN ILNDRNPVVT EDVEGDKKIY NAEVAEWDKL RQRLLDARNK
541 ITSAESAVNS ARNNVSARTN EQKHANDALN ALLKEKENIR SQLADINQKI AEEKRKRDEI
601 NMIKDAIKLT SDFYRTIYDE FGKQASELAK ELASVSQGKQ IKSVDDALNA FDKFRNNLNK
661 KYCLSYDTEI LTVEYGILPI GKIVEKRIEC TVYSVDNNGN IYTQPVAQWH DRGEQEVFEY
721 CLEDGCLIRA TKDHKFMTVD GQMMPIDEIF ERELDLMRVD NLPNGTAAND ENYALAA
```

Colicin E6

Mature amino acid sequence (WT)

```
  1 MSGGDGRGHN TGAHSTSGNI NGGPTGLGVG GGASDGSGWS SENNPWGGGS GSGIHWGGGS
 61 GHGNGGGNGN SGGGSGTGGN LSAVAAPVAF GFPALSTPGA GGLAVSISAG ALSAAIADIM
121 AALKGPFKFG LWGVALYGVL PSQIAKDDPN MMSKIVTSLP ADDITESPVS SLPLDKATVN
181 VNVRVVDDVK DERQNISVVS GVPMSVPVVD AKPTERPGVF TASIPGAPVL NISVNNSTPA
241 VQTLSPGVTN NTDKDVRPAG FTQGGNTRDA VIRFPKDSGH NAVYVSVSDV LSPDQVKQRQ
301 DEENRRQQEW DATHPVEAAE RNYERARAEL NQANEDVARN QERQAKAVQV YNSRKSELDA
361 ANKTLADAIA EIKQFNRFAH DPMAGGHRMW QMAGLKAQRA QTDVNNKQAA FDAAAKEKSD
421 ADAALSSAME SRKKKEDKKR SAENKLNEEK NKPRKGVKDY GHDYHPDPKT EI
```

SIML amino acid sequence

```
  1 MIKIATRKYL GKQNVYDIGV ERYHNFALKN GFIASNSGTG GNLSAVAAPV AFGFPALSTP
 61 GAGGLAVSIS AGALSAAIAD IMAALKGPFK FGLWGVALYG VLPSQIAKDD PNMMSKIVTS
121 LPADDITESP VSSLPLDKAT VNVNVRVVDD VKDERQNISV VSGVPMSVPV VDAKPTERPG
181 VFTASIPGAP VLNISVNNST PAVQTLSPGV TNNTDKDVRP AGFTQGGNTR DAVIRFPKDS
241 GHNAVYYVSVS DVLSPDQVKQ RQDEENRRQQ EWDATHPVEA AERNYERARA ELNQANEDVA
301 RNQERQAKAV QVYNSRKSEL DAANKTLADA IAEIKQFNRF AHDPMAGGHR MWQMAGLKAQ
361 RAQTDVNNKQ AAFDAAAKEK SDADAALSSA MESRKKKEDK KRSAENNLND EKNKPRKGFK
421 DYGHDYHPAP KTENIKGLGD LKPGIPKTPK QNGGGKRKRW TGDKGRKIYE WDSQHGELEG
481 YRASDGQHLG SFDPKTGNQL KGPDPKRNIK KYLMSGGDGR GHNTGAHSTS GNINGGPTGL
541 GVGGGASDGS GWSSENNPWG GGSGSGIHWG GGSGHGNGGG NGNSGGGCLS YDTEILTVEY
601 GILPIGKIVE KRIECTVYSV DNNGNIYTQP VAQWHDRGEQ EVFEYCLEDG CLIRATKDHK
661 FMTVDGQMMP IDEIFERELD LMRVDNLPNG TAANDENYAL AA
```

Colicin S4

Mature amino acid sequence (WT)

```
  1 MAKELSGYGP TAGESVGGTG ANLNQQGGNN NSNSGVHWGG GSGSGNGGGE HGSQTGWGWS
 61 KTNNPDVPPY VDDNGQVRIT ITNGLVKTPV YGVPGAGGNS DVQGGYIPEN PNDEVARKWD
121 KNNLPREIDV SIDGFKYRVT LNDNGRAIGI LRTGVRPYVG SEKAKAGIME KINHKTPEEI
181 YEALGFNKDE SQRQEKAKQQ AEDAWDNLPP NVRKFDVDVE QFHYLVVLDD YGNVLSVTRT
241 GVRPYVGSEK AKAGIMDKVD HKTPEEIYEA LGFNNEEPQR QNQAKKAAYD VFYSFSMNRD
301 RIQSDILNKS AEVISDIGNK VGDYLGDAYK SLAREIADDV KNFQGKTIRS YDDAMASLNK
361 VLSNPGFKLN RADSDALANA WRSIDAQDMA NKLGNFSKAF KFADVVMKVE KVREKSIEGY
421 ETGNWGPLML EVESWVLSGI ASAVALGVFS ATLGAYALSL GAPAIAVGIV GILLAAVVGA
481 LIDDKFADAL NNEIIKPAH
```

SIML amino acid sequence

```
  1 MIKIATRKYL GKQNVYDIGV ERYHNFALKN GFIASNSFSM NRDRIQSDIL NKSAEVISDI
 61 GNKVGDYLGD AYKSLAREIA DDVKNFQGKT IRSYDDAMAS LNKVLSNPGF KLNRADSDAL
121 ANAWRSIDAQ DMANKLGNFS KAFKFADVVM KVEKVREKSI EGYETGNWGP LMLEVESWVL
181 SGIASAVALG VFSATLGAYA LSLGAPAIAV GIVGILLAAV VGALIDDKFA DALNNEIIKP
241 AHMAKELSGY GPTAGESVGG TGANLNQQGG NNNSNSGVHW GGGSGSGNGG GEHGSQTGWG
301 WSKTNNPDVP PYVDDNGQVR ITITNGLVKT PVYGVPGAGG NSDVQGGYIP ENPNDEVARK
361 WDKNNLPREI DVSIDGFKYR VTLNDNGRAI GILRTGVRPY VGSEKAKAGI MEKINHKTPE
421 EIYEALGFNK DESQRQEKAK QQAEDAWDNL PPNVRKFDVD VEQFHYLVVL DDYGNVLSVT
481 RTGVRPYVGS EKAKAGIMDK VDHKTPEEIY EALGFNNEEP QRQNQAKKAA YDVFYCLSYD
541 TEILTVEYGI LPIGKIVEKR IECTVYSVDN NGNIYTQPVA QWHDRGEQEV FEYCLEDGCL
601 IRATKDHKFM TVDGQMMPID EIFERELDLM RVDNLPNGTA ANDENYALAA
```

Colicin D

Mature amino acid sequence

```
  1 MSDYEGSGPT EGIDYGHSMV VWPSTGLISG GDVKPGGSSG IAPSMPPGWG DYSPQGIALV
 61 QSVLFPGIIR RIILDKELEE GDWSGWSVSV HSPWGNEKVS AARTVLENGL RGGLPEPSRP
121 AAVSFARLEP ASGNEQKIIR LMVTQQLEQV TDIPASQLPA AGNNVPVKYR LMDLMQNGTQ
181 YMAIIGGIPM TVPVVDAVPV PDRSRPGTNS APVSPNLPDL VLSVGQMNTP VLSNPEIQEE
241 GVIAETGNYV EAGYTMSSNN HDVIVRFPEG SDVSPLYIST VEILDSNGLS QRQEAENKAK
301 DDFRVKKEEA VARAEAEKAK AELFSKAGVN QPPVYTQEMM ERANSVMNEQ GALVLNNTAS
361 SVQLAMTGTG VWTAAGDIAG NISKFFSNAL EKVTIPEVSP LLMRISLGAL WFHSEEAGAG
421 SDIVPGRNLE AMFSLSAQML AGQGVVIEPG ATSVNLPVRG QLINSNGQLA LDLLKTGNES
```

481 IPAAVPVLNA VRDTATGLDK ITLPAVVGAP SRTILVNPVP QPSVPTDTGN HQPVPVTPVH
541 TGTEVKSVEM PVTTITPVSD VGGLRDFIYW RPDAAGTGVE AVYVMLNDPL DSGRFSRKQL
601 DKKYKHAGDF GISDTKKNRE TLTKFRDAIE EHLSDKDTVE KGTYRREKGS KVYFNPNTMN
661 VVIIKSNGEF LSGWKINPDA DNGRIYLETG EL

SIML amino acid sequence

1 MIKIATRKYL GKQNVYDIGV ERYHNFALKN GFIASNSAPV SPNLPDLVLS VGQMNTPVLS
61 NPEIQEEGVI AETGNYVEAG YTMSSNNHDV IVRFPEGSDV SPLYISTVEI LDSNGLSQRQ
121 EAENKAKDDF RVKKEEAVAR AEAEKAKAEL FSKAGVNQPP VYTQEMMERA NSVMNEQGAL
181 VLNNTASSVQ LAMTGTGVWT AAGDIAGNIS KFFSNALEKV TIPEVSPLLM RISLGALWFH
241 SEEAGAGSDI VPGRNLEAMF SLSAQMLAGQ GVVIEPGATS VNLPVRGQLI NSNGQLALDL
301 LKTGNESIPA AVPVLNAVRD TATGLDKITL PAVVGAPSRT ILVNPVPQPS VPTDTGNHQP
361 VPVTPVHTGT EVKSVEMPVT TITPVSDVGG LRDFIYWRPD AAGTGVEAVY VMLNDPLDSG
421 RFSRKQLDKK YKHAGDFGIS DTKKNRETLT KFRDAIEEHL SDKDTVEKGT YRREKGSKVY
481 FNPNTMNVVI IKSNGEFLSG WKINPDADNG RIYLETGELM SDYEGSGPTE GIDYGHSMVV
541 WPSTGLISGG DVKPGGSSGI APSMPPGWGD YSPQGIALVQ SVLFPGIIRR IILDKELEEG
601 DWSGWSVSVH SPWGNEKVSA ARTVLENGLR GGLPEPSRPA AVSFARLEPA SGNEQKIIRL
661 MVTQQLEQVT DIPASQLPAA GNNVPVKYRL MDLMQNGTQY MAIIGGIPMT VPVVDAVPVP
721 DRSRPGTNCL SYDTEILTVE YGILPIGKIV EKRIECTVYS VDNNGNIYTQ PVAQWHDRGE
781 QEVFEYCLED GCLIRATKDH KFMTVDGQMM PIDEIFEREL DLMRVDNLPN GTAANDENYA
841 LAA

Colicin M

Mature amino acid sequence

1 METLTVHAPS PSTNLPSYGN GAFSLSAPHV PGAGPLLVQV VYSFFQSPNM CLQALTQLED
61 YIKKHGASNP LTLQIISTNI GYFCNADRNL VLHPGISVYD AYHFSKPAPS QYDYRSMNMK
121 QMSGNVTTPI VALAHYLWGN GAERSVNIAN IGLKISPMKI NQIKDIIKSG VVGTFPVSTK
181 FTHATGDYNV ITGAYLGNIT LKTEGTLTIS ANGSWTYNGV VRSYDDKYDF NASTHRGVIG
241 ESLTRLGAMF SGKEYQILLP GEIHIKESGK R

SIML amino acid sequence

1 MIKIATRKYL GKQNVYDIGV ERYHNFALKN GFIASNCLQA LTQLEDYIKK HGASNPLTLQ
61 IISTNIGYFC NADRNLVLHP GISVYDAYHF SKPAPSQYDY RSMNMKQMSG NVTTPIVALA
121 HYLWGNGAER SVNIANIGLK ISPMKINQIK DIIKSGVVGT FPVSTKFTHA TGDYNVITGA
181 YLGNITLKTE GTLTISANGS WTYNGVVRSY DDKYDFNAST HRGVIGESLT RLGAMFSGKE
241 YQILLPGEIH IKESGKRMET LTVHAPSPST NLPSYGNGAF SLSAPHVPGA GPLLVQVVYS
301 FFQSPNMCLS YDTEILTVEY GILPIGKIVE KRIECTVYSV DNNGNIYTQP VAQWHDRGEQ
361 EVFEYCLEDG CLIRATKDHK FMTVDGQMMP IDEIFERELD LMRVDNLPNG TAANDENYAL
421 AA

Salmocin E1B

Mature amino acid sequence

1 MSDNTIAYYE DGVPYSADGQ VVIVIDGKMP VDTGAGGTGG GGGGKVGGTS ESSAAIHATA
61 KWSKAQLQKS LEEKAARERE TAAAMAAAKA KRDALTQHLK DIVNDVLRYN ASRTPSATDL
121 AHANNMAMQA EAQRLGRAKA EEKARKEAEA AEKSLQEAER QREEAARQRA EAERQLKQAE
181 AEEKRLAALS EEARAVEITQ KNLAAAQSEL SKMDGEIKSL NVRLSTSIHA RDAEMNSLSG
241 KRNELAQESA KYKELDELVK KLEPRANDPL QNRPFFDATS RRARAGDTLA EKQKEVTASE
301 TRINELNTEI NQVRGAISQA NNNRNLKVQQ VTETENALKV AIDNLNSSQM KNAVDATVSF
361 YQTLTAKYGE KYSLIAQELA EQSKGKKISN VDEALAAFEK YKDVLDKKFS KADRDAIVNA

421 LKSVDYADWA KHLDQFSRYL KISGRVSTGY DIYSDIRKGM DTNDWRPLFL TLEKLAVDAG
481 VGYIVALGFS VIASTALGIW GVAIITGVIC SFVDKKDLEK LNEALGI

SIML amino acid sequence

1 MIKIATRKYL GKQNVYDIGV ERYHNFALKN GFIASNCSFV DKKDLEKLNE ALGIMSDNTI
61 AYYEDGVPYS ADGQVVIVID GKMPVDTGAG GTGGGGGGKV GGTSESSAAI HATAKWSKAQ
121 LQKSLEEKAA RERETAAAMA AAKAKRDALT QHLKDIVNDV LRYNASRTPS ATDLAHANNM
181 AMQAEAQRLG RAKAEEKARK EAEAAEKSLQ EAERQREEAA RQRAEAERQL KQAEAEEKRL
241 AALSEEARAV EITQKNLAAA QSELSKMDGE IKSLNVRLST SIHARDAEMN SLSGKRNELA
301 QESAKYKELD ELVKKLEPRA NDPLQNRPFF DATSRRARAG DTLAEKQKEV TASETRINEL
361 NTEINQVRGA ISQANNNRNL KVQQVTETEN ALKVAIDNLN SSQMKNAVDA TVSFYQTLTA
421 KYGEKYSLIA QELAEQSKGK KISNVDEALA AFEKYKDVLD KKFSKADRDA IVNALKSVDY
481 ADWAKHLDQF SRYLKISGRV STGYDIYSDI RKGMDTNDWR PLFLTLEKLA VDAGVGYIVA
541 LGFSVIASTA LGIWGVAIIT GVICLSYDTE ILTVEYGILP IGKIVEKRIE CTVYSVDNNG
601 NIYTQPVAQW HDRGEQEVFE YCLEDGCLIR ATKDHKFMTV DGQMMPIDEI FERELDLMRV
661 DNLPNGTAAN DENYALAA

Microcin V

Mature amino acid sequence (WT)

1 ASGRDIAMAI GTLSGQFVAG GIGAAAGGVA GGAIYDYAST HKPNPAMSPS GLGGTIKQKP
61 EGIPSEAWNY AAGRLCNWSP NNLSDVCL

SIML amino acid sequence

1 MIKIATRKYL GKQNVYDIGV ERYHNFALKN GFIASNCNWS PNNLSDVCLA SGRDIAMAIG
61 TLSGQFVAGG IGAAAGGVAG GAIYDYASTH KPNPAMSPSG LGGTIKQKPE GIPSEAWNYA
121 AGRLCLSYDT EILTVEYGIL PIGKIVEKRI ECTVYSVDNN GNIYTQPVAQ WHDRGEQEVF
181 EYCLEDGCLI RATKDHKFMT VDGQMMPIDE IFERELDLMR VDNLPNGTAA NDENYALAA

Aureocin A53

Mature amino acid sequence (WT)

1 MSWLNFLKYI AKYGKKAVSA AWKYKGKVLE WLNVGPTLEW VWQKLKKIAG L

SIML amino acid sequence

1 MIKIATRKYL GKQNVYDIGV ERYHNFALKN GFIASNSWLN FLKYIAKYGK KAVSAAWKYK
61 GKVLEWLNVG PTLEWVWQKL KKIAGLMCLS YDTEILTVEY GILPIGKIVE KRIECTVYSV
121 DNNGNIYTQP VAQWHDRGEQ EVFEYCLEDG CLIRATKDHK FMTVDGQMMP IDEIFERELD
181 LMRVDNLPNG TAANDENYAL AA

Enterocin A

Mature amino acid sequence (WT)

1 TTHSGKYYGN GVYCTKNKCT VDWAKATTCI AGMSIGGFLG GAIPGKC

SIML amino acid sequence

1 MIKIATRKYL GKQNVYDIGV ERYHNFALKN GFIASNSIGG FLGGAIPGKC TTHSGKYYGN
61 GVYCTKNKCT VDWAKATTCI AGMCLSYDTE ILTVEYGILP IGKIVEKRIE CTVYSVDNNG
121 NIYTQPVAQW HDRGEQEVFE YCLEDGCLIR ATKDHKFMTV DGQMMPIDEI FERELDLMRV
181 DNLPNGTAAN DENYALAA

Enterocin HF

Mature amino acid sequence (WT)

```
  1 KYYGNGVSCN KKGCSVDWGK AIGIIGNNAA ANLTTGGKAG WKG
```

SIML amino acid sequence

```
  1 MIKIATRKYL GKQNVYDIGV ERYHNFALKN GFIASNSCNK KGCSVDWGKA IGIIGNNAAA
 61 NLTTGGKAGW KGKYYGNGVC LSYDTEILTV EYGILPIGKI VEKRIECTVY SVDNNGNIYT
121 QPVAQWHDRG EQEVFEYCLE DGCLIRATKD HKFMTVDGQM MPIDEIFERE LDLMRVDNLP
181 NGTAANDENY ALAA
```

Hiracin JM79

Mature amino acid sequence (WT)

```
  1 ATYYGNGLYC NKEKCWVDWN QAKGEIGKII VNGWVNHGPW APRR
```

SIML amino acid sequence

```
  1 MIKIATRKYL GKQNVYDIGV ERYHNFALKN GFIASNCWVD WNQAKGEIGK IIVNGWVNHG
 61 PWAPRRATYY GNGLYCNKEK CLSYDTEILT VEYGILPIGK IVEKRIECTV YSVDNNGNIY
121 TQPVAQWHDR GEQEVFEYCL EDGCLIRATK DHKFMTVDGQ MMPIDEIFER ELDLMRVDNL
181 PNGTAANDEN YALAA
```

Supplementary Data S3. Amino acid sequences of the split intein constructs used for the modular assembly of colicin-derived bacteriocins. In all constructs, the Npu DnaE split intein fragments (NpuN and NpuC) are shaded in grey to distinguish them from the bacteriocin-derived domains.

<u>Colicin K (T and R domains) – NpuN</u>

```
  1 MAKELSGYGP TAGESMGGTG ANLNQQGGNN NSNSGVHWGG GSGHGNNGGQ GNSNSSGSTS
 61 TVMKTGESYL TPWGDVVINN DGLPVMNGIV MTEENSTLVD NPFGGVSRVL NSLISDMPSL
121 FAESSGNNNN NTASVNTAPT NAQVSDMDKS SKVVSNVINE KQKQKNKIAT QISEKQKKIE
181 EMKKVFKHHS YHGITDLERD VDELQKKSNQ LDADISKLNS YKNTLQSKIG DVNKQKEAEE
241 KARENAEVAE HETLNEEKQA VAEAEKRLAE AKAELAKAES DVQSKQATVS RVAGELENAQ
301 KSVDVKVTGF PGWRDVQKKL QRQLEAKQAE YSAVENELKN AVSFRDGKAA EVKEAEQKLK
361 EAQDALEKCL SYDTEILTVE YGILPIGKIV EKRIECTVYS VDNNGNIYTQ PVAQWHDRGE
421 QEVFEYCLED GCLIRATKDH KFMTVDGQMM PIDEIFEREL DLMRVDNLPN
```

<u>Salmocin E1B (T and R domains) - NpuN</u>

```
  1 MSDNTIAYYE DGVPYSADGQ VVIVIDGKMP VDTGAGGTGG GGGGKVGGTS ESSAAIHATA
 61 KWSKAQLQKS LEEKAARERE TAAAMAAAKA KRDALTQHLK DIVNDVLRYN ASRTPSATDL
121 AHANNMAMQA EAQRLGRAKA EEKARKEAEA AEKSLQEAER QREEAARQRA EAERQLKQAE
181 AEEKRLAALS EEARAVEITQ KNLAAAQSEL SKMDGEIKSL NVRLSTSIHA RDAEMNSLSG
241 KRNELAQESA KYKELDELVK KLEPRANDPL QNRPFFDATS RRARAGDTLA EKQKEVTASE
301 TRINELNTEI NQVRGAISQA NNNRNLKVQQ VTETENALKV AIDNLNSCLS YDTEILTVEY
361 GILPIGKIVE KRIECTVYSV DNNGNIYTQP VAQWHDRGEQ EVFEYCLEDG CLIRATKDHK
421 FMTVDGQMMP IDEIFERELD LMRVDNLPN
```

<u>NpuC – Colicin K (C domain)</u>

```
  1 MIKIATRKYL GKQNVYDIGV ERYHNFALKN GFIASNSQIK DAVDTMVGFY QYITEQYGEK
 61 YAKIAQDLAE KSKGKKIQGV DEALAAFEKY KNVLDKKFSK VDRDAIFNAL ESVNYDELSK
121 NLTKISKSLK ITSRVSFLYD VGSDFKNAIE TGNWRPLFVT LEKSAVDVGV AKIVALMFSF
181 IVGVPLGFWG IAIVTGIVSS YIGDDELSKL NELLGI
```

<u>NpuC – Salmocin E1B (C domain)</u>

```
  1 MIKIATRKYL GKQNVYDIGV ERYHNFALKN GFIASNSQMK NAVDATVSFY QTLTAKYGEK
 61 YSLIAQELAE QSKGKKISNV DEALAAFEKY KDVLDKKFSK ADRDAIVNAL KSVDYADWAK
121 HLDQFSRYLK ISGRVSTGYD IYSDIRKGMD TNDWRPLFLT LEKLAVDAGV GYIVALGFSV
181 IASTALGIWG VAIITGVICS FVDKKDLEKL NEALGI
```

Supplementary Data S4. Amino acid sequences of the constructs used in the inducible SIML system. The Npu DnaE split intein fragments (NpuN and NpuC) are highlighted in grey, whereas the Cat split intein fragments (CatN and CatC) are highlighted in red. The remaining residues correspond to the mature enterocin AS-48 sequence.

NpuC – EntAS48 – NpuNA – CatN

```
  1 MIKIATRKYL GKQNVYDIGV ERYHNFALKN GFIASNSLLA AAGRESIKAY LKKEIKKKGK
 61 RAVIAWMAKE FGIPAAVAGT VLNVVEAGGW VTTIVSILTA VGSGGLCLSY DTEILTVEYG
121 ILPIGKIVEK RIECLSGDTM IEILDDDGII QKISMEDLYQ RLA
```

CatC - NpuNB

```
  1 MFKLNTKNIK VLTPSGFKSF SGIQKVYKPF YHHIIFDDGS EIKCSDNHPF GKDKIKASTI
 61 KVGDYLQGKK VLYNEIVEEG IYLYDLLNVG EDNLYYTNGI VSHNCTVYSV DNNGNIYTQP
121 VAQWHDRGEQ EVFEYCLEDG CLIRATKDHK FMTVDGQMMP IDEIFERELD LMRVDNLPNG
181 T
```

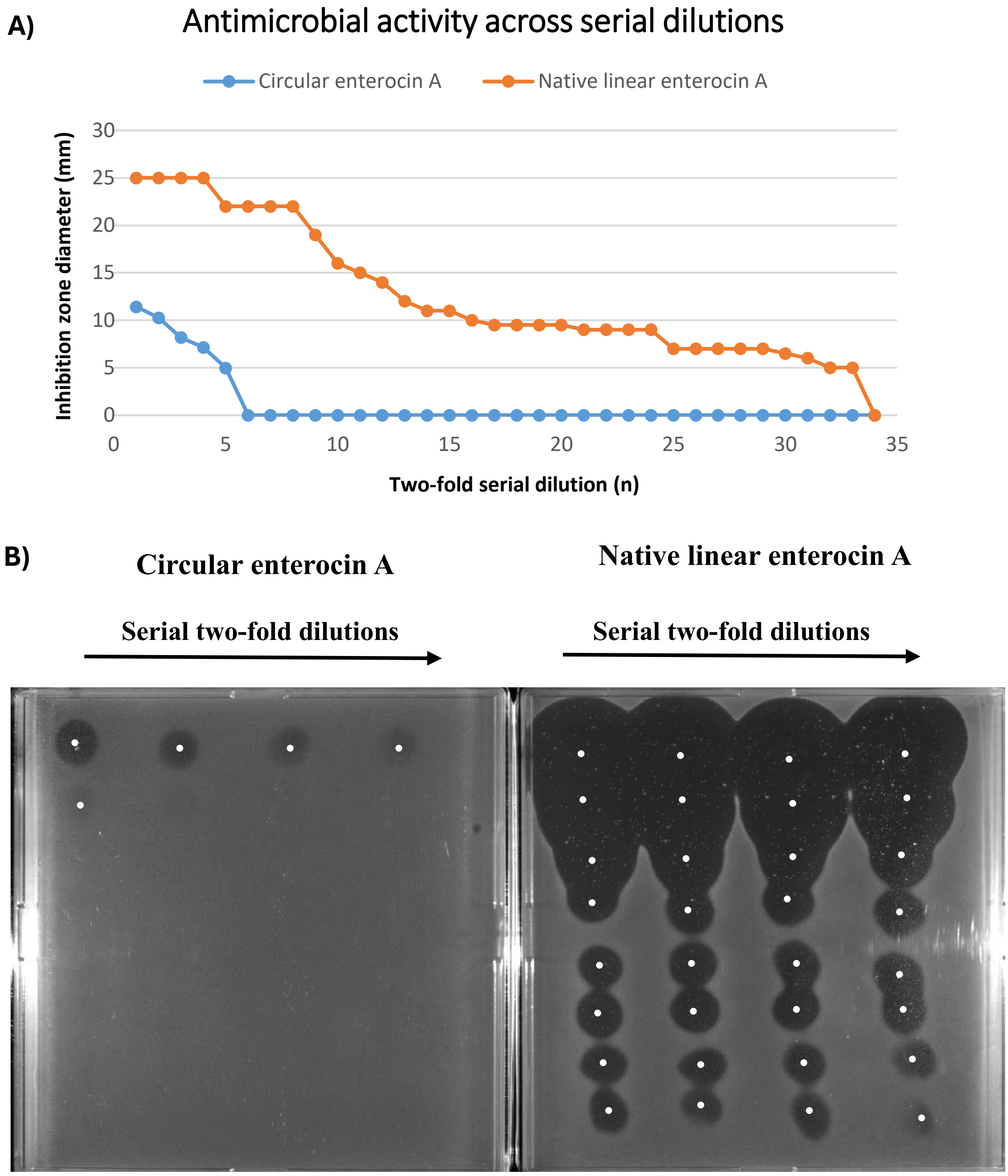

Figure S1. Comparative antimicrobial activity of chemically synthesized backbone-circularized and native linear enterocin A across serial two-fold dilutions. (A) Inhibition zone diameters produced by chemically synthesized backbone-circularized enterocin A (blue) and native linear enterocin A (orange) following serial two-fold dilutions. (B) Antimicrobial activity of the backbone-circularized (left) and native linear (right) enterocin A variants, evaluated by the spot-on-agar test (SOAT) against *Listeria monocytogenes* CECT 4032. Two-fold serial dilutions were applied sequentially from left to right and continued on the subsequent row.
